# Proportion of night eating syndrome in Arab population of Oman

**DOI:** 10.1186/s40337-015-0079-6

**Published:** 2015-11-25

**Authors:** Fahad Zadjali, Aaisha Al-Bulushi, Fatma AlHassani, Mustafa Al Hinai

**Affiliations:** Department of Biochemistry, College of Medicine and Health Sciences, Sultan Qaboos University, P.O box 35, 123 Muscat, Oman; College of Medicine and Health Sciences, Sultan Qaboos University, Muscat, Oman; Department of Family Medicine and Public Health, College of Medicine and Health Sciences, Sultan Qaboos University, Muscat, Oman

**Keywords:** Night eating, Obesity, Middle East, Evening hyperphagia

## Abstract

**Objective:**

Globally obesity has reached epidemic proportions with alarming rates in the Arabian Gulf countries. The impact of behavioral eating habits and in particular night eating syndrome (NES) have not been emphasized in the region. This study assessed the proportion of NES in an Omani Arab adult population sample.

**Method:**

A night eating syndrome questionnaire (NEQ) was distributed to Omani adults above the age of 20.

**Results:**

Out of the 454 respondents, 26.4 % endorsed evening hyperphagia while nocturnal ingestion was present in 4.7 % of the respondents. In addition, 1.5 % of the respondents met the NES criteria.

**Conclusions and implications:**

The proportion of NES in Omani adult population is similar to the reported rates in general world populations. In conclusion, night eating syndrome is present in the Omani Arab adults and should be taken into account in national management for increased obesity trends in the region.

Obesity is a condition of high morbidity and a major concern in the Arabian Gulf region with prevalence ranging between 15 and 48 % in adults [1–3] and 20.5 % in Oman [4]. The rising trend of obesity leads to social and economical challenge to the Omani government.

Night eating syndrome (NES) is an example of delayed circadian intake of food that was first noted in obese individuals [5]. It is mainly characterized by evening hyperphagia and nocturnal awakening with ingestion of food. The estimated prevalence of night eating syndrome in the general population is 1.5 %, 15 % among obese individuals and 42 % among morbid obese individuals [6, 7]. The extent of NES in the Middle East region has not been studied. The purpose of this study is to estimate the proportion of NES in a general Omani adult population sample.

## Methods

A previously published and validated NES questionnaire (NEQ) was used to assess night eating [8]. Questions were divided into 4–5 Likert scales and both Arabic and English translations were provided (Table 1). The NEQ was distributed online to staff and students of Sultan Qaboos University. Respondent were selected based on age ≥ 20 years and Omani nationality. The study protocol was approved by the University ethics committee (code: MREC#632). Data were analyzed using SPSS software (version 19) and questions were tested for internal validity using Cronbach’S alpha test with item deletion.

**Table 1:** Items of night eating syndrome questionnaires (NEQ)

Item #	Question
1	How often do you feel that you eating at night more than morning and afternoon?
2	When was your first meal in the day?
3	How is your appetite in the morning?
4	Do you have trouble in getting into sleep?
5	Number of times per night you get awake while sleeping.
6	Do you eat when you wake up at night?
7	Do you eat to make yourself sleepy before bedtime?
8	Do you control your eating in the evening time?

## Results

Out of 454 responded-participants (mean age 33 ± 5), 231 (50.9 %) were men and 223 (49.1 %) were women. The NEQ has a maximum score of 35 and individuals with night eating syndrome were defined using the standard NEQ cut-off point of 25 and a trend toward NES when NEQ score is between 20 and 24 [9]. Cronbach’s alpha reliability for the total scale was 0.5.

The average score of NEQ was 17.3 ± 3.2 in the study population. Seven individuals met the criteria of NES (1.5 %, 95 % C.I: 0.8–3.2) and 92 individuals (20.3 %, 95 % C.I: 16.8–24.2) showed a trend toward NES. There was no significant gender difference between the NES groups (Chi-square P-value 0.326). Proportion of NES was higher in men (2.2 %) compared to 0.9 % in women. Based on self-report from the NEQ, 26.4 % of the sample endorsed evening hyperphagia while nocturnal ingestion was present in 4.7 % of the respondents. Morning anorexia was present in 26 % of the respondents.

## Discussion

This study assessed night eating syndrome among Omani adults using an online version of the NEQ. Within the study population, 1.5 % met criteria for night eating syndrome, which is similar to international general population prevalence of 0.5–1.5 % [6].

The high prevalence of overweight and obesity among Arab countries is attributed to multiple factors including rapid nutrition transition, high socioeconomic status, physical inactivity and genetic admixtures [10, 11]. Eating behavior and circadian rhythm are proving to be important factors in the etiology of obesity. In obese individuals NES is more frequent and the majority of patients undergoing bariatric surgery may have NES [6]. Inclusion of self reported body mass index was a limitation in the study and association between NES and obesity was not assessed. This limitation suggests the need for further exploration of the NES in both obese and non-obese individuals in Arabs.

In conclusion, the proportion of night eating syndrome in Omani adults is similar to its prevalence in general population. Night eating syndrome maybe preventable among obese patient and it should be considered in management of obesity.

## References

[1] Badran M, Laher I (2011). Obesity in arabic-speaking countries. J Obes.

[2] Rahim HF, Sibai A, Khader Y, Hwalla N, Fadhil I, Alsiyabi H (2014). Non-communicable diseases in the Arab world. Lancet.

[3] AL S (2014). Obesity in gulf countries. Int J Health Sci.

[4] Al-Lawati JA, Jousilahti PJ (2004). Prevalence and 10-year secular trend of obesity in Oman. Saudi Med J.

[5] Marshall HM, Allison KC, O’Reardon JP, Birketvedt G, Stunkard AJ (2004). Night eating syndrome among nonobese persons. Int J Eat Disord.

[6] Rand CS, Macgregor AM, Stunkard AJ (1997). The night eating syndrome in the general population and among postoperative obesity surgery patients. Int J Eat Disord.

[7] Stunkard AJ, Allison KC (2003). Two forms of disordered eating in obesity: binge eating and night eating. Int J Obes Relat Metab Disord.

[8] Allison KC, Lundgren JD, O’Reardon JP, Martino NS, Sarwer DB, Wadden TA (2008). The Night Eating Questionnaire (NEQ): psychometric properties of a measure of severity of the Night Eating Syndrome. Eat Behav.

[9] Lundgren JD, Allison KC, Crow S, O’Reardon JP, Berg KC, Galbraith J (2006). Prevalence of the night eating syndrome in a psychiatric population. Am J Psychiatry.

[10] Musaiger AO, Al-Mannai M, Tayyem R, Al-Lalla O, Ali EY, Kalam F (2012). Prevalence of Overweight and Obesity among Adolescents in Seven Arab Countries: A Cross-Cultural Study. J Obes.

[11] Zadjali F, Al-Yahyaee S, Hassan MO, Albarwani S, Bayoumi RA (2013). Association of adiponectin promoter variants with traits and clusters of metabolic syndrome in Arabs: family-based study. Gene.

